# Surface roughness data of microsphere-based coatings developed to counter the urban heat island phenomenon

**DOI:** 10.1016/j.dib.2025.112061

**Published:** 2025-09-15

**Authors:** Silvia Cavagnoli, Claudia Fabiani, Chiara Chiatti, Anna Laura Pisello

**Affiliations:** a EAPLAB at CIRIAF, Interuniversity Research Center, University of Perugia, via G. Duranti 63, 06125 Perugia, Italy; b Department of Engineering, University of Perugia, via G. Duranti 93, 06125 Perugia, Italy

**Keywords:** Profilometer, Photosimulation, Height parameters, Hybrid parameters, Steel grit, Ceramic microspheres

## Abstract

This work presents a dataset derived from the characterization and surface roughness analysis of coatings developed for the mitigation of the urban heat island phenomenon. In detail, these coatings were produced using a black painted aluminium layer with the surface application of ceramic microspheres and chrome steel grit, both in three different sizes, i.e. a small, a medium, and a large one. The aim of the dataset is to demonstrate how the different types of material applied influences the surface topography and affects roughness in comparison to a reference sample. The analysis was carried out with the NANOVEA Jr25 profilometer, which allowed the collection of useful data for the evaluation of the main surface parameters and the precise measurement of the height and distribution of micro-irregularities. The adopted approach ensured the accurate representation of the surface microstructure, and the dataset obtained was processed with the Mountains8 software to obtain height and hybrid parameters. Analysing the dataset in detail, the samples with ceramic microspheres show a lower roughness than those with steel grit. This reduction in roughness may contribute to improved optical and radiative properties of the surfaces, making ceramic microspheres suitable for radiative cooling applications and mitigation of the urban heat island effect.

Specifications Table

**Table:** 

Subject	Engineering & Materials science
Specific subject area	Optical profilometry of microsphere materials for characterizing the surface roughness of innovative reflective coatings for urban heat island mitigation
Type of data	Images (.png), Text (.txt), Raw data (.csv)
Data collection	The surface roughness of the samples was analyzed with the NANOVEA Jr25 chromatic confocal profilometer. Scans were taken over an area of 1 × 1 cm with a CL4 lens and MG35 magnifying glass. The data, processed with Mountains8 software, provided height (ISO 25,178) and hybrid (EUR 15,178 N) parameters. Roughness was isolated by means of Gaussian filters: an S-filter (2.5 µm) and an l-filter (0.8 mm), to remove micro-roughness and undulations.
Data source location	EAPLAB at CIRIAF, Interuniversity research Centre, University of Perugia, Perugia, Italy
Data accessibility	Repository name: Zenodo Data identification number: 10.5281/zenodo.15807697 Direct URL to data: https://doi.org/10.5281/zenodo.15807697
Related research article	S. Cavagnoli, C. Fabiani, C. Chiatti, A. L. Pisello, Optimizing surface performance for urban heat island mitigation using microsphere-based coatings. Under Review, Sustainable Energy Technologies and Assessments SETA-D–25–00141R3

## 1 Value of the Data

• The profilometric data are representative of microscale surface alterations induced by ceramic microspheres and steel grit on black-based aluminum substrates, under controlled conditions and with high spatial resolution (1 cm × 1 cm scans). These data provide 2D and 3D topographical characterizations of surface textures using standardized parameters (ISO 25178, EUR 15178 N).
• These data provide a quantitative understanding of how different microsphere sizes and materials affect surface roughness and texture complexity. The values of height (Sa, Sq, Sp, Sv, etc.) and hybrid parameters (Sdq, Sdr) allow for detailed comparisons between the various finishes, which are crucial in applications where surface morphology influences functional performance (e.g. solar reflectance).
• The adopted methodology, based on Chromatic Confocal technology and robust two-step filtering (S and L filters), ensures reproducibility and minimizes noise, making the data reliable for surface engineering studies and texture-based functional analyses.
• The collected data can be used as a benchmark to compare the effectiveness of different surface treatments where surface roughness plays a key role.
• Profilometric maps and parameters offer high spatial resolution and are surface-specific, allowing an analysis of surface heterogeneity. This approach can be particularly useful for studying localised wear or controlled texturing in surface design.
• This research is of fundamental importance to the scientific community working on urban heat island mitigation through the development of innovative materials and to those combining radiative cooling properties with roughness. Furthermore, material manufacturers and industries could use this data to evaluate the key roughness parameters of final products.

## 2 Background

The urban heat island effect (UHI) represents one of the most pressing environmental challenges in metropolitan areas, where surface temperatures can be significantly higher than in surrounding rural areas due to human activity, infrastructure construction, and declining vegetation, compromising overall city liveability [[1], [2]]. To counteract this phenomenon, passive cooling via cool materialas [[3], [4]] and, more in general, radiative cooling technologies have gained increasing interest, as it offers a method to reduce temperatures without the need for external energy input. However, this technology is still under development and faces several challenges related to material preparation and dependence on weather conditions. Based on this, some recent approaches have attempted to overcome these limitations, drawing inspiration from natural phenomena and analyzing the mechanisms of certain animals [5]. Based on a study of the Cyphochilus beetle, the study by Xu et al. [6] developed a material that combines radiative and evaporative cooling using a porous polydimethylsiloxane sponge incorporating the hygroscopic salt LiCl. In addition to high reflectivity and emissivity in the atmospheric window, these materials can improve cooling efficiency thanks to this dual functionality. Again inspired by nature, the study by Xu et al. [7] focused on the nanostructure of the wings of the Pieris rapae butterfly, which served as inspiration for the development of a double-layer polymer film. Again, this material demonstrated high solar reflectivity and high thermal emissivity in the atmospheric window.

In the context of these innovations and the development of novel mechanisms for innovative radiative cooling materials, this work focuses on the study of micromaterial-induced surface roughness in radiative coatings. In particular, microspheres have been shown to enhance radiative cooling properties, but the correlation between surface morphology and cooling performance remains poorly explored. The dataset developed in this study aims to fill this gap by characterizing the surface roughness of the materials.

In particular, the dataset was generated to characterize the changes in surface roughness induced by the application of ceramic and steel microspheres on black-based substrates. The main motivation was to provide a standardized, high-resolution analysis of how different microspheres sizes and materials affect surface morphology. The study is based on profilometric analysis performed with a NANOVEA Jr25 profilometer equipped with confocal chromatic technology, which allows non-contact, high-precision 3D surface measurements. Data processing followed internationally recognized standards, using a two-stage filtering approach (S and L filters with robust Gaussian filters) to isolate relevant roughness scales and exclude shape and waviness components. This data set is associated with an original research article investigating the morphological and functional effects of microsphere-based surface treatments on key thermo-optical properties. This data article complements the research article by providing complete data sets, 3D surface reconstructions, photosimulations, height and hybrid parameters for surface characterization, and surface roughness data. This type of analysis allows the scientific community to integrate these data together with other characterization parameters and see how one affects the other.

## 3 Data Description

From the profilometric analysis of the samples, a 1 × 1 cm scan of the analysed sample surface was obtained. The processed image for each sample is called “Surface” (.png) in the data repository. In a similar manner, a photosimulation and 3D view of the analysed sample part was also obtained (i.e. called “Photosimulation” (.png) and “3D” (.png) in the data repository). A section was then extracted from the scanned surface, the data of which are presented in the “Section” file (.txt), with the first column that refers to the x axis in µm and the second column that refers to the z axis in µm. From the profilometric analysis of the samples, height parameters (Sq, Ssk, Sku, Sp, Sv, Sa) and hybrid parameters (Sdq and Sdr) were calculated and analysed. In particular, the parameters are defined as follows:

• Sq – Root-mean-square height: standard deviation of the height distribution;
  $$Sq=\sqrt{\frac{1}{A}\int \;\;\;\int_{A}\;z^{2}\left(x,y\right)dxdy}$$
• Ssk – Skewness: a negative value suggests that the surface is predominantly characterized by deep valleys, whereas a positive value indicates a surface with numerous peaks;
  $$Ssk=\frac{1}{Sq^{3}}\left[\frac{1}{A}\int \;\;\;\int_{A}\;z^{3}\left(x,y\right)dxdy\right]$$
• Sku – Kurtosis: a value <3 indicates that the height distribution is skewed with respect to the mean plane, =3 the height distribution is normal, >3 the height distribution is sharp;
  $$Sku=\frac{1}{Sq^{4}}\left[\frac{1}{A}\int \;\;\;\int_{A}\;z^{4}\left(x,y\right)dxdy\right]$$
• Sp – Maximum peak height: height between the highest peak and the mean plane;
• Sv – Maximum pit height: depth between the mean plane and the deepest valley;
• Sz – Maximum height: height between the highest peak and the deepest valley;
• Sa – Arithmetic mean height: mean surface roughness;
  $$Sa=\frac{1}{A}\int_{A}\;\left|z\left(x,y\right)\right|dxdy$$
• Sdq – Root-mean-square slope of the surface: mean quadratic slope of the surface roughness. It is a parameter that measures how much a surface is sloped on average with respect to its area;
  $$Sdq=\sqrt{\frac{1}{A}\int \;\;\;\int_{A}\;\left[{\left(\frac{\partial z\left(x,y\right)}{\partial x}\right)}^{2}+{\left(\frac{\partial z\left(x,y\right)}{\partial y}\right)}^{2}\right]dxdy}$$
• Sdr – Developed interfacial area ratio: complexity of the surface. If the value is equal to 0 it means that the surface is completely flat, when the irregularity of the surface increases, also Sdr increases.
  $$Sdr=\frac{1}{A}\left[\int \;\;\;\int_{A}\;\left(\sqrt{\left[1+{\left(\frac{\partial z\left(x,y\right)}{\partial x}\right)}^{2}+{\left(\frac{\partial z\left(x,y\right)}{\partial y}\right)}^{2}\right]}-1\right)dxdy\right]$$

## 4 Experimental Design, Materials and Methods

Seven samples were produced as part of this study, each consisting of a 5 × 5 cm aluminium base [8] painted with black acrylic spray paint [9]. One of the seven samples produced was kept without further treatment and used as a reference (REF), while the remaining six were modified by the addition of ceramic microspheres [10] and steel grit [11] of different sizes applied over a transparent adhesive layer to ensure uniform distribution and adhesion.

**Fig. 1:**
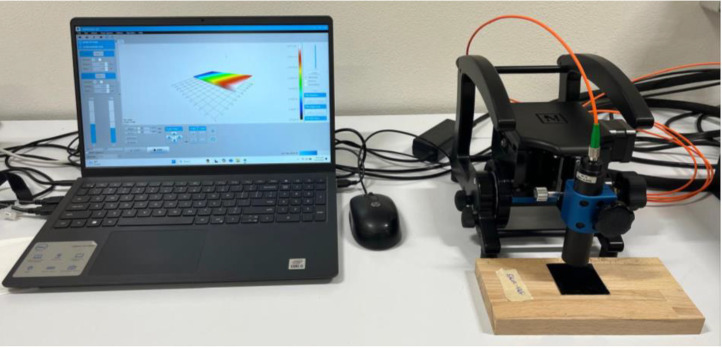
Profilometric analysis setup.

The surface height and roughness parameters of the samples were analysed with the NANOVEA Jr25 profilometer (Fig. 1), which operates with Chromatic Confocal technology. This technology uses the wavelengths of light to accurately determine the surface height.

For this study, black-based samples with ceramic microspheres and steel grit were examined in three different sizes: ceramic in small (from 0 to 50 µm), medium (from 125 to 250 µm), and big (from 425 to 600 µm) sizes and steel grit in small (50 µm), medium (from 150 to 250 µm), and big (from 400 to 600 µm) sizes. More in detail, the ceramic microspheres have a glass structure of 68 % Zirconium and 32 % vitreous phase. Chemical analysis of the materials reports ZrO_2_= 60–70 %, SiO_2_= 28–33 %, AL_2_O_3_ <10 %, apparent density = 2.30 kg/l, specific weight = 3.85 g/cm^3^, and hardness = 50–65 HRC. The steel grit is instead composed as follows: *C* = Max 0.3 %, Cr ≈ 14 %, Ni ≈ 1 %, Hardness = 42 HRC. Each sample was scanned over an area of 1 cm × 1 cm using an optical pen equipped with a CL4 chromatic lens and an MG35 magnifying glass. The data obtained were processed with Mountains8 software, extracting surface height parameters (ISO 25,178 [12]) and hybrid parameters (EUR 15,178 N [13]).

To accurately analyse the roughness of the samples, it was necessary to remove micro-roughness, waviness, and shape irregularities. This was achieved using a two-step filtering process:

• An S-filter with a Robust Gaussian filter of order 2 and a cut-off of 2.5 µm was applied to eliminate small-scale components below this value (short wavelengths), effectively isolating micro-roughness.
• An L-filter with a Robust Gaussian filter of order 2 and a cut-off of 0.8 mm was used to remove large-scale components above this value (long wavelengths), refining the focus on medium-scale roughness.

**Fig. 2:**
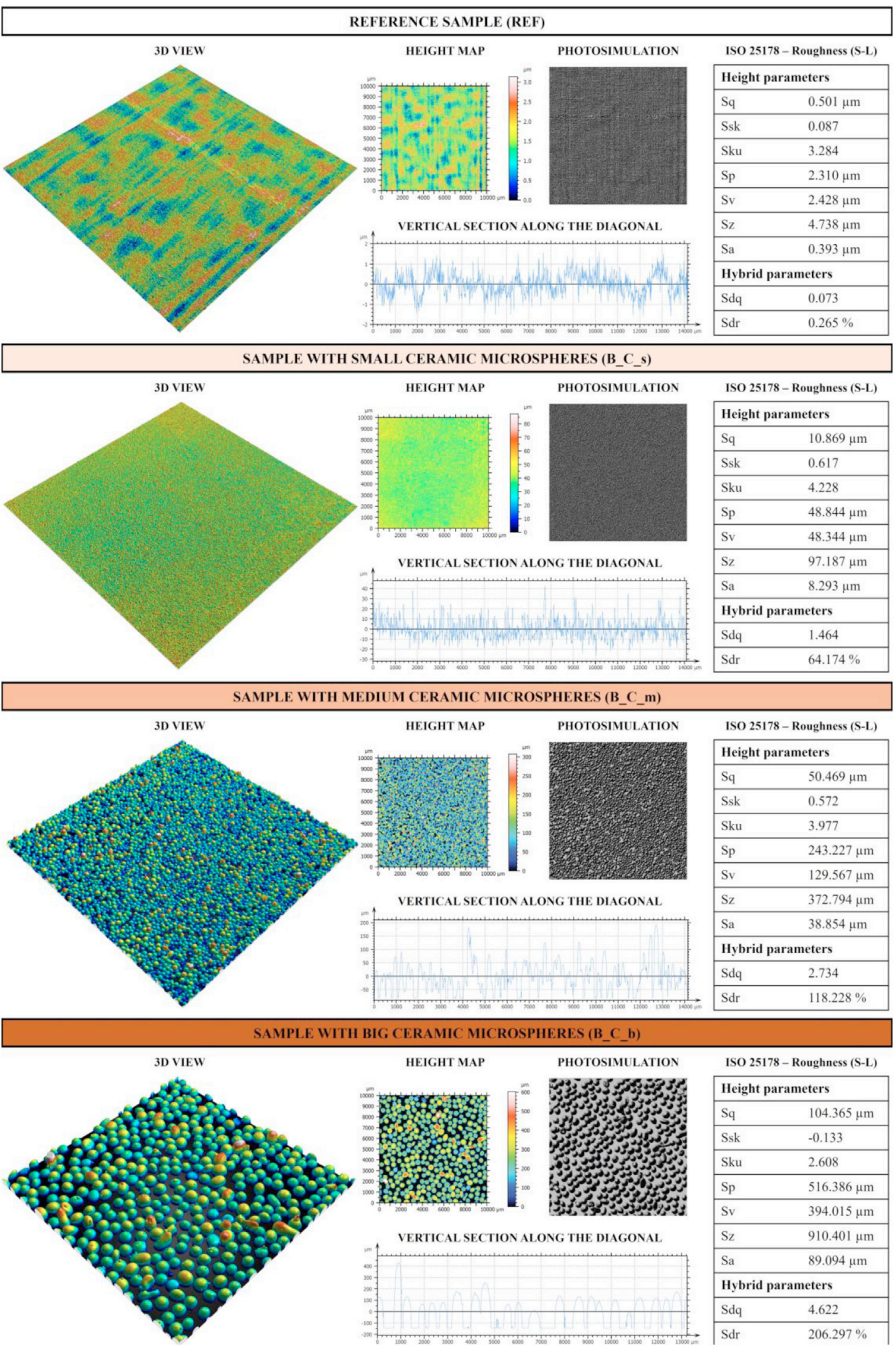
Profilometric analysis on B_REF and samples with ceramic microspheres.

**Fig. 3:**
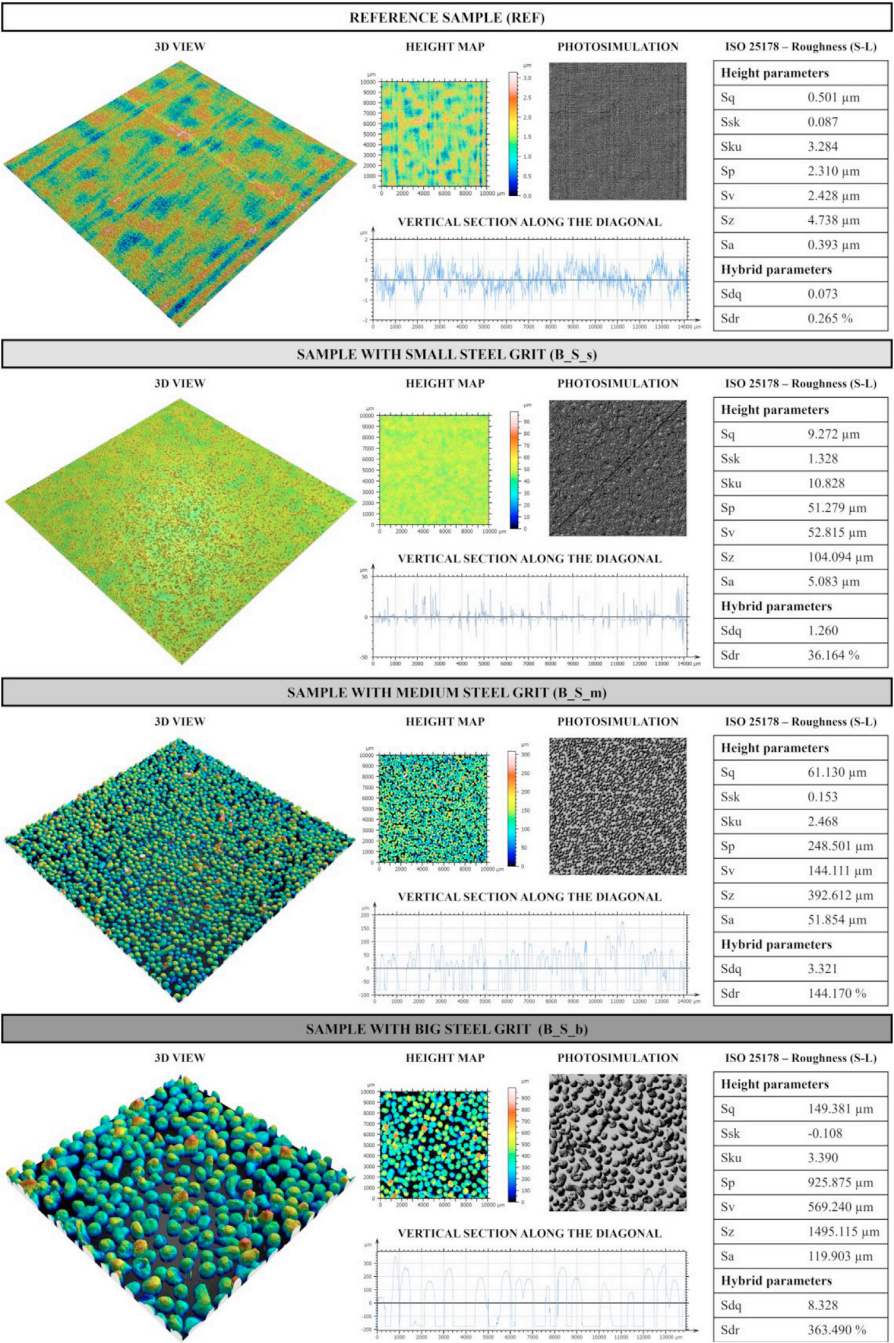
Profilometric analysis on B_REF and samples with steel grit.

For each sample, the 3D view, the height map recorded in the 1 cm × 1 cm scan, the photosimulation, a section along the diagonal, and the calculated parameters are presented. Fig. 2 shows the results of profilometric analysis on B_REF and samples with ceramic microspheres, while Fig. 3 shows the results of profilometric analysis on B_REF and samples with steel grit.

When analysing the reference sample (REF), it is evident that the surface has minimal roughness. The Sq parameter has a relatively low value, in line with the Sa value of 0.393 µm, indicating a smooth surface. The Ssk value is close to 0, suggesting that the height distribution is symmetrical around the mean, while the Sku value is nearly 3, indicating a normal distribution of surface heights. Although the surface is mostly smooth, small irregularities are present, as indicated by the parameters Sp, Sv and Sz. The Sdq value further confirms the smoothness of the surface, indicating that the average slope of the surface roughness is relatively low, meaning that the roughness is not particularly steep. In addition, the Sdr value of 0.265 % indicates that only 0.265 % of the surface consists of peaks or asperities, while the rest consists of largely flat areas.

All these parameters increase with the application of microspheres, both with ceramic and steel ones. In particular, by analysing the Sq and Sa parameters of samples with microspheres, it is evident that the application of steel grit generally leads to higher roughness. However, for the smallest size, ceramic microspheres produce the highest level of roughness. This may be due to the microspheres’ different chemical compositions and shapes as ceramic microspheres are spherical while steel grit is spheroidal, and this may have influenced the roughness. Furthermore, the steel grit applied has a size of 50 µm, while ceramic ones are between 0 and 50 µm. Therefore, in the sample with ceramic microspheres analysed there are more microspheres of different sizes that have influenced the final roughness value. However, for medium and larger microspheres the roughness is higher for the steel grit samples. Their spheroidal shape covered the application area differently, creating greater roughness in the scanned area analysed. Consequently, the height parameters Sp, Sv, and Sz also increase with the introduction of microspheres, increasing as the size of the applied microspheres increases. In contrast, the Ssk parameter is always positive, except for large microspheres in both ceramic and steel. This also decreases as the size of the microspheres increases. This is consistent, in that given the larger size of the microspheres, the distribution of heights is skewed toward the higher part of the graph, characteristic of an Ssk<0. A negative Skewness value indicates that the surface has more valleys than peaks, meaning that the valleys are deeper and sharper than the peaks, which are smoother. Larger microspheres have greater heights and raise the mean reference plane, resulting in more valleys, which is why the parameter is negative. The distribution is biased toward valleys, which are more numerous or depper. As for the value of Sku, it is generally greater than 3, indicating a distribution of heights with sharp peaks (sharp trend), except in the cases of B_C_b and B_S_m, where the value is slightly <3, indicating a distribution with a more obtuse trend.

As for the hybrid parameters, Sdq increases as the size of the microspheres increases. This indicates an increase in the average slope of the surface asperities. Thus, larger microspheres tend to generate a surface with more pronounced peaks and valleys, making the surface less flat and more irregular. As the size of the microspheres increases, the surface then becomes steeper and more irregular, with more pronounced asperities. This is related to the increase in SDR value as the size of the microspheres increases. Indeed, an increase in SDR means that the density and percentage of surface area covered by asperity increases as the size of the applied microspheres increases. Therefore, it means that the surface is becoming rougher, with a higher asperity density, which is indeed also related to the calculated Sa values.

Therefore, the application of microspheres, especially larger microspheres, significantly transforms surfaces by making them rougher, more complex, and characterized by more asperities. This effect is amplified using larger microspheres, which generate surfaces with more pronounced slopes and more asymmetrical height distributions. In addition, ceramic and steel microspheres exhibit distinct behaviours, offering different options for modulating surface properties according to specific needs.

## Limitations

• The profilometric analysis was performed on a limited area (1 cm × 1 cm). This approach may not capture the macroscopic heterogeneity of the surface or may not be fully representative, despite the special care taken to homogeneously distribute the microspheres in this case during sample production. To verify the representativeness of the 1 cm x 1 cm scan, 4 areas were extracted from this scan and the various parameters were analyzed. The coefficient of variation (CV) was calculated for the Sq and Sa parameters, which are the fundamental parameters for this analysis, by calculating it as the ratio between the standard deviation and the average multiplied by 100. Table 1 reports the CV values of the selected parameters for all samples.

**Table 1:** Coefficient of variation.

COEFFICIENT OF VARIATION		
	Sq	Sa
REF	4.089	3.952
B_C_s	2.410	3.554
B_C_m	4.403	3.809
B_C_b	2.869	2.228
B_S_s	3.878	7.328
B_S_m	3.503	3.106
B_S_b	4.074	2.225

• The application of the microspheres was done manually. This may result in uncontrolled variations in the density, distribution and orientation of the microspheres, especially at the microscopic level, affecting the roughness parameters obtained. In addition, some microspheres could be stacked on top of each other.

## Ethics Statement

The authors have read and followed the ethical requirements for publication in Data in Brief and confirm that this work does not involve human subjects, animal experiments or data collected from social media platforms.

## CRediT authorship contribution statement

**Silvia Cavagnoli:** Data curation, Investigation, Methodology, Software, Validation, Visualization, Writing – original draft. **Claudia Fabiani:** Conceptualization, Data curation, Formal analysis, Investigation, Methodology, Software, Supervision, Validation, Visualization, Writing – review & editing. **Chiara Chiatti:** Data curation, Investigation, Methodology, Visualization. **Anna Laura Pisello:** Conceptualization, Formal analysis, Funding acquisition, Methodology, Project administration, Resources, Supervision, Validation, Writing – review & editing.

## Data Availability

zenodoOptical profilometry of microsphere materials for characterizing the surface roughness of innovative reflective coatings for urban heat island mitigation (Original data).

## Appendix Supplementary materials

Image, application 1
